# Malignant Transformation of Rat Kidney Induced by Environmental Substances and Estrogen

**DOI:** 10.3390/ijerph9051630

**Published:** 2012-05-04

**Authors:** Susana Alfaro-Lira, María Pizarro-Ortiz, Gloria M. Calaf

**Affiliations:** 1 Instituto de Alta Investigación, Universidad de Tarapacá, Calle Antofagasta 1520, Arica, Chile; Email: saalfaro@uta.cl (S.A.-L.); marililia24@hotmail.com (M.P.-O.); 2 Center for Radiological Research, Columbia University Medical Center, 630 West 168th Street, New York, NY 10032, USA

**Keywords:** estrogen, malathion, kidney, atypical cell proliferation, MFG, ER-α, ER-β, PgR, CYP1A1, Neu/ErbB2, PCNA, vimentin, THBS

## Abstract

The use of organophosphorous insecticides in agricultural environments and in urban settings has increased significantly. The aim of the present study was to analyze morphological alterations induced by malathion and 17β-estradiol (estrogen) in rat kidney tissues. There were four groups of animals: control, malathion, estrogen and combination of both substances. The animals were injected for five days and sacrificed 30, 124 and 240 days after treatments. Kidney tissues were analyzed for histomorphological and immunocytochemical alterations. Morphometric analysis indicated that malathion plus estrogen-treated animals showed a significantly (*p* < 0.05) higher grade of glomerular hypertrophy, signs of tubular damage, atypical proliferation in cortical and hilium zone than malathion or estrogen alone-treated and control animals after 240 days. Results indicated that MFG, ER-α, ER-β, PgR, CYP1A1, Neu/ErbB2, PCNA, vimentin and Thrombospondin 1 (THB) protein expression was increased in convoluted tubules of animals treated with combination of malathion and estrogen after 240 days of 5 day treatment. Malignant proliferation was observed in the hilium zone. In summary, the combination of malathion and estrogen induced pathological lesions in glomeruli, convoluted tubules, atypical cell proliferation and malignant proliferation in hilium zone and immunocytochemical alterations in comparison to control animals or animals treated with either substance alone. It can be concluded that an increased risk of kidney malignant transformation can be induced by exposure to environmental and endogenous substances.

## 1. Introduction

The interaction of chemical carcinogens with healthy cells associated with exogenous hormones can induce genomic damage and subsequently cause cancer with the ability of metastasize other tissues [1,2,3,4]. The carcinogenesis process needs several mutational events to produce damage to the genome, and subsequent cell proliferation of these injured cells. DNA damage can be the result of interactions with exogenous agents such as chemical carcinogens [1,2]. The organophosphorous pesticides are chemical substances synthesized by men and mainly used for pest control in agriculture [5,6,7] and residential urban surroundings [8,9]. The use of such pesticides such as malathion has increased significantly due to its low mammalian toxicity, short persistence in the environment, broad spectrum of activity and low cost. However, no studies have determined the real impact of this use. The commercial or technical grade of malathion used for pest control is not a pure molecule and it is associated with impurities produced by manufacture or storage [10,11]. Reports have clearly indicated that commercial or technical grade malathion caused oxidative damage symptoms *in vitro* and *in vivo* in male rats after four weeks of malathion exposure, such as decreased number of spermatogenic cells or necrosis in seminiferous tubules and edema in interstitial tissues [12]. Different studies have shown that malathion and other pesticides induced histopathological alterations in the kidneys of experimental animals. These changes included parenchymatous degeneration of the cells of renal tubules and hyperemia of the cortical part of the kidney, especially of renal glomeruli [13]. 

Human milk fat globule (MGF) is abundant in breast milk and is synthesized by epithelial cells of differentiated mammary glands. The MGF is made up of proteins, triglycerides and phospholipids which are encapsulated by membranes of the apical portion of these mammary cells. MGF membranes are composed by many glycoproteins which serve as markers for differentiated carcinomas [14]. The MFG-E8 (milk fat globule-EGF factor 8) also known as p47, lactadeherin, rAGS, PAS6/7 and BA-46 , is a glycoprotein expressed in many tissues including mammary gland and is expressed and often over-expressed on the surface of breast carcinoma cells. [14,15]

The estrogen 17β-estradiol is an endogenous hormone present in women that influences development, control of ovulation, implantation, fertilization and metabolism of minerals, carbohydrates, proteins and lipids. The production of endogenous estrogen ceases with menopause and there is a controversy concerning its use in hormone replacement therapy. There is strong epidemiological and clinical evidence that estrogens play a role in the induction, promotion and progression of a variety of cancers in target organs of rat, mice and hamster [16,17]. Other studies have associated estrogen administration to postmenopausal women with an increased risk of endometrial and breast cancer [18,19,20]. Estrogens are also associated with several cancers in humans and are known to induce kidney tumors in rodents [21]. 

The estrogen receptors (ER) are members of the nuclear receptors superfamily and specifically to the family of steroid receptors that function as ligand-regulated transcription factors [22]. ER, including ER-α and ER-β are expressed in a variety of normal and malignant tissues, regulating a variety of physiological functions in animals and human body [23]. The expression patterns of the clinically most critical molecules for breast cancer are ER, as well as progesterone receptor (PgR) and Neu/ErbB-2/ HER2. The Neu/ErbB-2 is a transmembrane glycoprotein with tyrosine kinase activity, which gene is present in normal cells as a single copy, but in malignant cells is amplified and over-expressed. This phenomenon has been seen in multiple human cancers, including carcinoma of breast, ovary, uterus, lung, kidney, stomach and pancreas [24]. In experimental studies the over-expression of Neu/ErbB-2 protein has been shown to be an important determinant of malignant transformation, development of metastatic disease, and increased cell proliferation [25].

Human cytochrome P450 (CYP) enzymes play a key role in the metabolism of drugs and environmental chemicals. The CYP1A-isoenzymes, CYP1A1 and CYP1A2, are primarily responsible for the metabolic activation the pro-carcinogens to genotoxic intermediates [26]. One of these isoforms, the cytochrome CYP1A1 present in extra hepatic tissues, has an aryl-hydrocarbon hydroxylase activity, and it is involved in the metabolic activation of several carcinogenic substances [27]. The proliferative cell nuclear antigen (PCNA) is a 36 kDa molecular weight protein also known as cyclin [28]. It has also been identified as the polymerase delta-associated protein [29]. It is synthesized in early G1 and S phases of the cell cycle [29,30]. Vimentin is a cytoskeletal intermediate filament protein type III, normally found in embrionic or mesenchymal originated cells. However it is frequently expressed in neoplastic cells with metastatic properties including breast cancer [31,32]. Thrombospondin-1, also called TBHS-1 or TSP-1, is an adhesive glycoprotein which has a role in cellular phenotype regulation during genesis and tissue repair by cell to cell and cell-matrix interaction [33]. Also TBHS-1 has been shown a participation in angiogenesis process and facilitation cellular migration of invasive breast cancer cells [34,35]. 

At present, there are no studies which have examined the effects of an organophosphorous pesticide such as malathion in combination with estrogen exposure in relation to increased risk of kidney transformation. The aim of this study was to examine the morphological alterations in the kidney tissue in animals that showed mammary gland cancer when exposed to malathion and 17β-estradiol 240 days after 5-day injections. The objective of this work was also to investigate the expression patterns of the most clinically critical molecules for breast cancer such as ER, PR and HER2, as well as others involved in cell cycle, angiogenesis and cellular migration of invasive breast cancer cells, frequently expressed in neoplastic cells with metastatic properties and enzymes that play a key role in the metabolism of drugs and environmental chemicals.

## 2. Experimental Section

### 2.1. Experimental Designs

There were four experimental groups of 39 day-old virgin female Sprague Dawley rats obtained from the Catholic University of Chile (Santiago, Chile). Nine animals per group were housed and bred in a barrier animal facility operated in accordance with the standards outlined in Guide for the Care and Use of Laboratory Animals [36]. All rats were allowed continuous access to a standard laboratory chow diet (Champion, Santiago, Chile). The animals were injected subcutaneously twice a day for five days with: physiological saline solution (control), 100 μg/100 g body weight (BW); malathion (M, Fyfanon TM, Cheminova, Denmark), 22 mg/100 g BW; 17β-estradiol (estrogen) (Sigma-Aldrich Chemical Co., Milwaukee, WI, USA), 30 μg/100 g BW; and combination of both treatments. The LD_50_ value for M was 1.000 mg/kg BW which allowed a 100% survival of animals after 5-day treatment. The doses used in these experiments were one sixth the LD_50_ for malathion, which allowed a 100% survival of animals after a 5-day treatment period. The animals were sacrificed in three periods after 30, 124 and 240 days post treatment and the kidney tissue were excised and morphologically analyzed. The rats were sacrificed under ether anesthesia and opened by a midline incision from the pubis to remove the kidneys. 

### 2.2. Morphological Studies

Rat kidney tissue was removed and fixed in 10% neutral buffered formalin for histopathological studies. The kidney tissue sections (five slides per sample) were oriented flat and sectioned at 5 µm thickness, deparaffinized and stained with hematoxylin eosin and all of them were evaluated under a light microscope. Throughout histological studies the severity of kidney damage was evaluated by a scoring system which gave quantitative measurements. Ten fields on the zone cortical were studied with a 10× lens in an optical microscope (Olympus CX31). One hundred glomeruli were observed and classified according to scale of glomerular hypertrophy from “1 to 4” points. Glomeruli radiuses that fluctuated from 43.77 to 59.73 µm were graded as “1”; from 59.74 to 73.96 µm “2”; from 73.97 to 88.19 µm as “3” and over 88.20 µm as “4” points. The tubular damage was analyzed in five sectors per animal and the totality of fields was analyzed for morphological abnormalities. The damage was quantified with score from “1 to 4”. Structures were graded as “1” when morphology and normal tubular structure were present and there was lack of hyaline casts, calcifications in tubules and hemorrhagic zones. Structures were graded as “2” when minor damage in cell morphology and tubular structures were present and there was not hyaline casts in kidney, with few calcifications in the tubules and few hemorrhage zones. Structures were graded as “3” when the cell morphology and tubular structure had damage in proximal tubules without microvilli and dilated tubules and there were small hyaline casts in several areas of the kidney, several calcifications in tubules and hemorrhagic zones. Structures were graded as “4” when the cell morphology and tubular structure had serious injuries and presence of large hyaline casts, hemorrhage zones and abundant amount of calcifications in the tubular zone. Five slides per animal were observed to analyze atypical cellular proliferations in the cortical area and hilium zone. Proliferation zones were classified into categories from 0 to 3 according to the number of proliferations found in the histological section. Zones without proliferation were graded as “0”, with one proliferation zone was graded as “1”, two to three zones as “2”, and four or more as “3”.

### 2.3. Immunocytochemical Staining

Protein expression was evaluated by peroxidase immunochemical staining. Tissues were incubated with 1% H_2_O_2_ in methanol for 30 min, in order to block endogenous peroxidase, washed twice with a buffer solution, and fixed with buffered paraformaldehyde in PBS, pH 7.4, at room temperature. Subsequently, tissues were then covered with normal serum for 30 min at room temperature. The slides were then washed once and incubated with the corresponding antibodies at a 1:500 dilution overnight at 4 °C. The following antibodies used were: MFG-E8 (MFG-06): SC-8029, mouse monoclonal antibody; ER-α (MC-20): SC-542, rabbit polyclonal antibody; ER-β (Y-19): SC-6821, goat polyclonal antibody; PgR (C-20) SC-538 rabbit polyclonal antibody; CYP1A1 (B-4) SC-25304 mouse monoclonal antibody; Neu/ErbB-2 (C-18): SC-284, rabbit polyclonal antibody; PCNA (PC10) SC-56, mouse monoclonal antibody; Vimentin (C-20) SC-7557 rabbit polyclonal antibody; thrombospondin 1 or TSP-1 (A4.1) SC-59886 mouse monoclonal antibody (all from Biotechnology Inc., Santa Cruz, CA, USA). Protein expression was determined by using the avidin-biotin-horseradish immunoperoxidase peroxidase complex (Standard ABC kit; Vector, Burlingame, CA, USA). The 3, 3c-Diaminobenzidine (DAB) (Sigma-Aldrich, St. Louis, MO, USA) was used as a chromogen. For negative controls, duplicate samples were immunostained without exposure to the primary antibody or substituted with pre-immune serum. 

### 2.4. Statistical Analysis

Statistical analysis was performed by using statistical package SPSS version 15.0 for Windows. Comparison between treated groups and controls were made by Anova and Dunnet’s test, with *p* < 0.05 and the test of hypothesis for difference of proportions (*p* < 0.05).

## 3. Results

**Figure 1:**
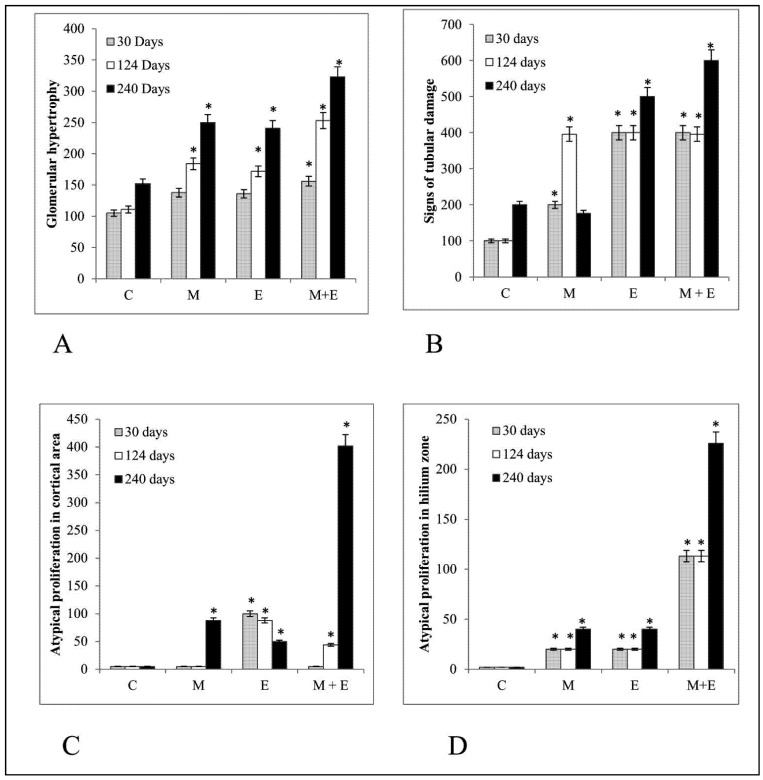
The graphs show the degree of (**A**) Glomerular hypertrophy, (**B**) Signs of tubular damage, Atypical proliferation in (**C**) Cortical area and (**D**) hilium zone. The error bars correspond to 5% error. (*) Significant difference (*p* < 0.05) between treatment and control group. (C) control, (M) malathion, (E) estrogen, (M + E) malathion in combination with estrogen.

The combination of malathion and estrogen induced greater changes in the tubular section of the kidneys in comparison with either substance alone. Figure 1 shows graphs of the different groups where the degree of changes are appreciated, such as glomerular hypertrophy (A), signs of tubular damage (B), atypical proliferation in cortical area (C) and atypical proliferation in hilium zone (D) after 30, 124 and 240 days post treatments. There were four groups of rats consisting of control, malathion, estrogen and combination of both substances. Sections from untreated rat kidneys showed normal glomeruli and convoluted tubules. The cortico-medulary junction and renal hilum had normal aspect since was lined by normal transitional uroepithelium after 30, 124 and 240 days post treatment. In Figure 1A shows the effects of malathion, estrogen alone and the combination of both on glomerular hypertrophy. Neither substance alone induced a significant increase. However, there was a significant (*p* < 0.05) increase in this parameter in the presence of both substances after 30 days of treatment. 

After the 124 days malathion treatment did not induce significant changes in relation to cell proliferation in the cortical area, but it caused several examples of certain damage in comparison with control, such as a significant (*p* < 0.05) increase in the degree of glomerular hypertrophy, signs of tubular damage and proliferation in the hilium zone. The same treatment caused more significant (*p* < 0.05) alterations after 240 days, evidenced by glomerular hypertrophy, signs of tubular damage, and atypical proliferation in the cortical and hilium zones in comparison with the control group.

Estrogen treatment did not cause changes in the glomerular hypertrophy, but it caused a significant (*p* < 0.05) increase in tubular damage in comparison with the control and malathion treatment groups. This was the only treatment that caused a significant (*p* < 0.05) increase in atypical proliferation in cortical area after 30 days post-treatment in comparison with control, malathion and the combination of malathion and estrogen treatments. 

Estrogen treatment did not cause changes in the proliferation of hilium after 30 days in comparison with control (Figure 1D). 

**Figure 2:**
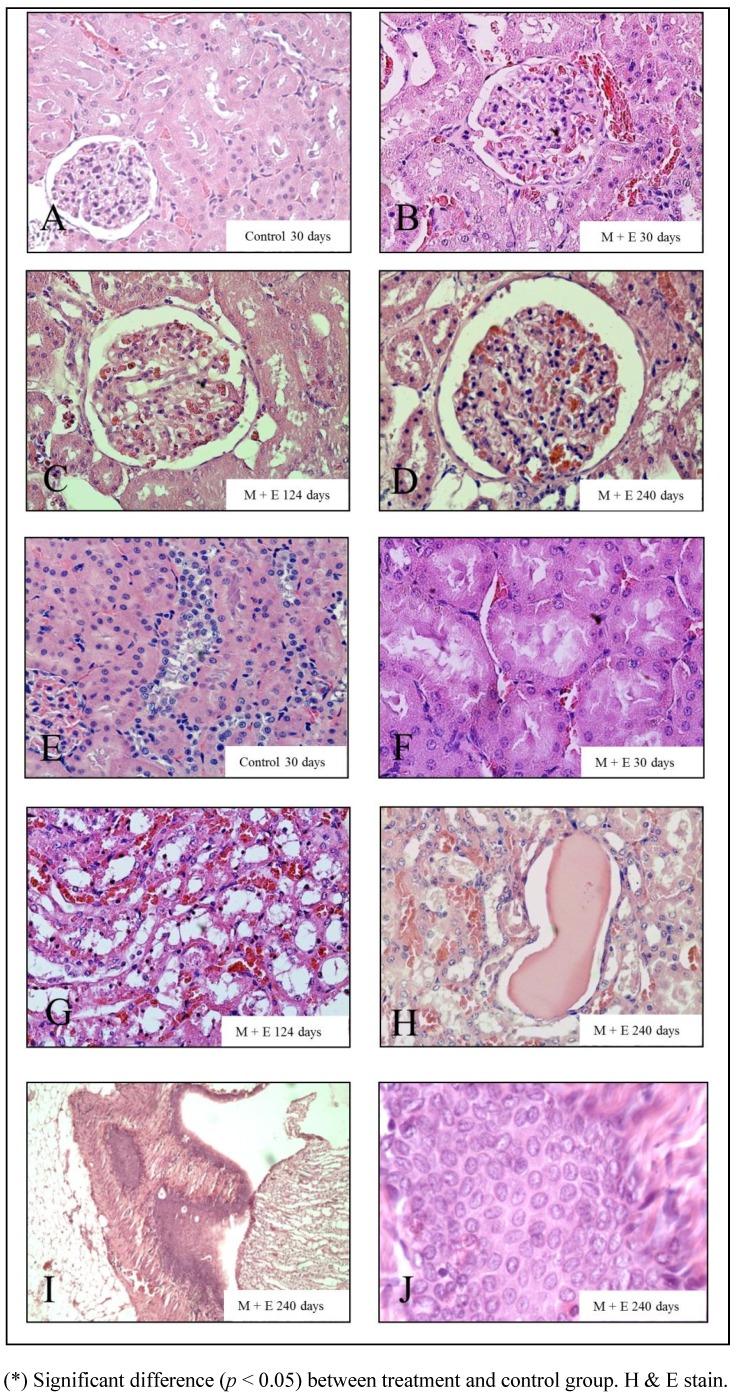
Representative images of glomerular hypertrophy graded from 1 a 4 points (**A–D**) and normal (**E**) and damaged tubular structures (**F–H**).

The histological examination showed that estrogen induced a significant (*p* < 0.05) increase in the degree of glomerular hypertrophy and tubular damage in comparison with control after 124 days. On the other hand, estrogen caused a significant (*p* < 0.05) increase of the atypical proliferation in the cortical area in comparison to all other treated animals after 124 days post-treatment. There was a significant (*p* < 0.05) increase in glomerular hypertrophy, tubular damage, atypical proliferation in the cortical and hilium areas in comparison with control after 240 days.

The combination of malathion and estrogen caused a significant (*p* < 0.05) increase in the glomerular hypertrophy, grade of tubular damage and degree of atypical proliferation in the hilium zone in comparison with control group, estrogen and malathion-treated animals by 30 days after treatment. The malathion plus estrogen-treated animals did not show an increase in proliferation in the cortical area after 30 days. This group displayed a significant (*p* < 0.05) glomerular hypertrophy in comparison with the malathion, estrogen and control groups after 124 and 240 days post-treatment. The grade of tubular damage was significantly (*p* < 0.05) higher in combined treatments in comparison with the estrogen and control groups after 124 and 240 days. It also caused a significant (*p* < 0.05) increase in the degree of proliferation in the cortical area in comparison with control after 124 days. The amount of atypical proliferation in the hilium zone found after malathion plus estrogen treatment was significantly (*p* < 0.05) greater in comparison to the malathion, estrogen and control groups after 124 and 240 days (Figure 1C and Figure 1D). The combination of both treatments caused in certain areas malignant proliferation in the hilium area by 240 days after treatment (Figure 2I–J).

Figure 2 corresponds to representative images of glomerular hypertrophy graded from 1 a 4 points (Figure 2A–D) and normal and damage tubular structure (Figure 2G–H). Grade 1 had normal radius between 43.77–59.73 µm; Grade 2 presented increased cellularity of the capillary tuft and radius between 59.74–73.96 µm; Grade 3 corresponded to increased number of cells in capillary tuft and thickness of the basement membrane, and radius between 73.97–88.19 µm; and (D) Grade 4 had radius over 88.20 µm. Representative images of tubular damage morphology with (E) normal tubular structure, (F) minor damage, (G) moderate damage in proximal tubules without microvilli and dilated tubules with hyaline casts inside (H) serious injury as abundant number of foci of hemorrhage, large hyaline casts (data not shows), amount of calcification of tubular, desquamation in to the lumen of the tubule cells and decrease in cell height. Representative figures of the tumor found in malathion plus estrogen-treated rat (Figure 2I) and 100× (Figure 2J) can also be seen.

**Figure 3:**
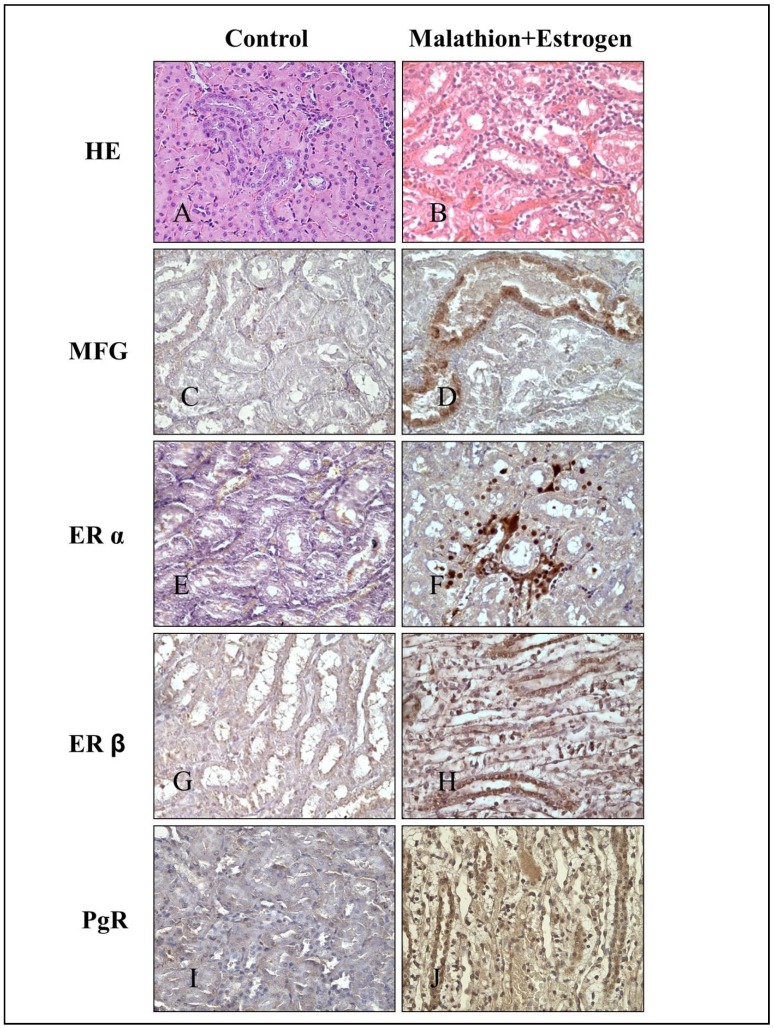
(**A–B**) Representative images of tubules stained with H & E (HE). Immunochemical staining of control and combination of malathion and estrogen-treated rats after 240 days of 5 days treatment. Protein expression of (**C–D**) MGF, (**E–F**) ERα, (**G–H**) ERß and (**I–J**) PgR.

**Figure 4:**
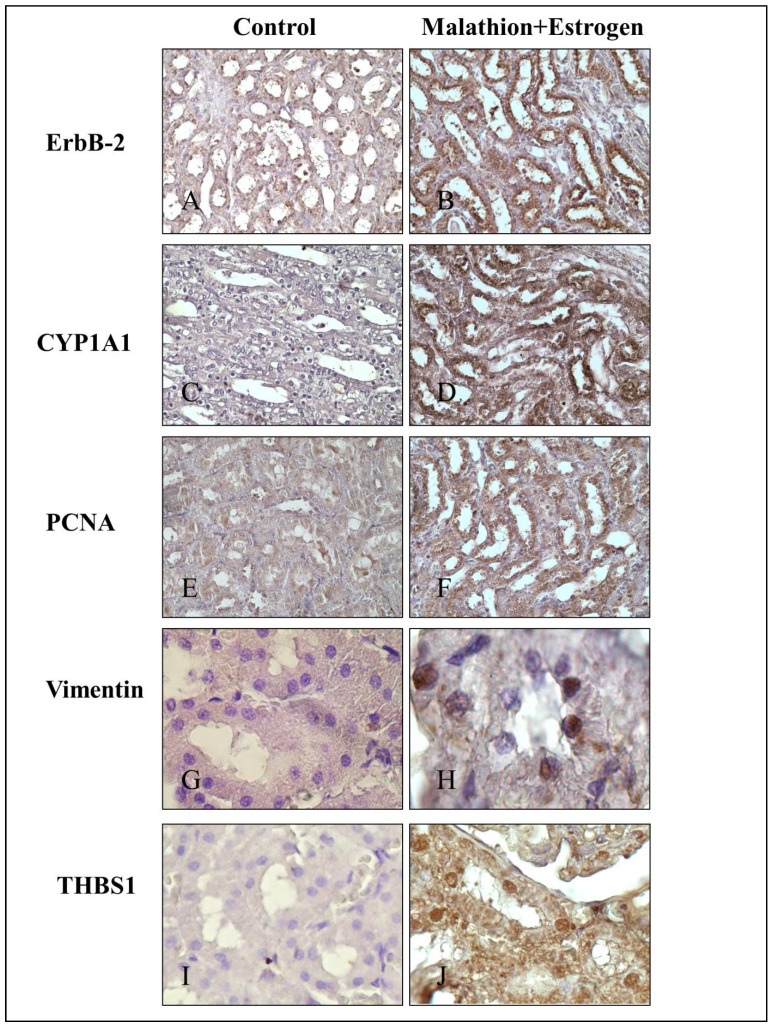
Representative images of immunochemical staining of control and combination of malathion and estroegen-treated rats after 240 days of 5 days treatment: Protein expression of (**A–B**) Neu/ErbB2, (**C–D**) CYP1A1, (**E–F**) PCNA, (**G–H**) vimentin and (**I–J**) thrombospondin 1.

Representative images of tubules stained with HE and immunochemical staining of control (A) and combination of malathion and estrogen-treated (B) rats 240 days after five day treatment can be seen in Figure 3. They correspond to HE (Figure 3A and 3B), MFG (Figure 3C and 3D), ERα (Figure 3E and 3F) ER β (Figure 3G and 3H), PgR and (Figure 3I, 3J and 3E), protein expression in tubules of kidney. Figure 4 corresponds to representative images of tubules. Immunochemical staining of control (A) and combination of malathion and estrogen-treated (B) rats 240 days after five day treatment. They correspond to ErbB-2 (Figure 4A and 4B) CYP1A1 (Figure 4C and 4D), PCNA (Figure 4E and 4F), Vimentin (Figure 4G and 4H), and THBS1 (Figure 4I and 4J), protein expression in kidney tubules. The combination of malathion and estrogen caused a significant increase in the protein expression of all these markers in comparison to controls.

## 4. Discussion

These studies showed that a combination of malathion and estrogen caused malignant phenotypic alterations in kidney tissues and the injuries increased progressively with time after the five day exposure that was greater than the effect of either substance alone and reaching mammary gland tumor formation after 240 days. The present study showed important morphohistological alterations in the kidneys of rats treated for five days with malathion, estrogen or a combination of both when examined 30, 124 and 240 days after treatments in comparison to controls. The alterations found were glomerular hypertrophy, signs of tubular damage, atypical proliferation convoluted tubules and atypical proliferation in the hilium zone. Those proliferative alterations in the cortical and hilium area were caused mainly by the presence of both substances, malathion and estrogen, in comparison to either substance alone. These abnormalities can be suggested as a sign of progression of malignancy. Furthermore, the association of both substances also caused a higher grade of glomerular hypertrophy in comparison to either substance alone. It has been suggested that glomerular hypertrophy is one most important non-immunological factors contributing to the development of glomerulosclerosis and may represent a sign of malignancy [37].

The combination of malathion and estrogen treatments produced greater tubular damage in comparison with these substances alone. Malathion alone caused a greater degree of damage in the tubular structures in comparison with estrogen-treatment. The presence of hyaline acts as a sign of tubular damage that could be explained by an elevation of urinary protein. Reports have indicated an increase of urinary protein in rats genetically predisposed to autoimmune disease and exposed to malathion [38]. An increase of urinary proteins were observed in presence of hyaline cast, as a sign of glomerular inflammation that is often associated with reduced renal function [39].

The toxic effects of commercial malathion can be explained by the presence of impurities in these commercial formulations, and these substances are metabolized in the liver and processed into more polar and toxic metabolites such as malaoxon, which is excreted by the kidneys causing tubular damage [40,41,42]. In other reports, animals exposed to acute treatment showed that the most sensitive targets of oxidative damage were the kidneys, lungs and diaphragm [43]. Investigations have revealed that malathion exposure was associated with necrosis and edema in the seminiferous tubules and interstitial tissues in male rats [44,45]. Other authors have demonstrated that chronic exposure to malathion produced signs of histological damage in the kidney marked by hyperplasia or hypertrophy of tubular cells and morphologic alterations in glomerulus [46]. Similar results were found in this study with acute exposure to malathion. These results suggest that acute and chronic exposure to malathion can produce long-term human health consequences. Numerous studies in humans have indicated that there is an elevated risk of developing cancers such as non-Hodgkins lymphoma and leukemia in farmers exposed to malathion and other chemicals used in agriculture [47,48,49].

Estrogen induced several injuries in kidneys such as atypical proliferation in the cortical area that started after 30 days and increased up to 240 days. In a male hamster model, the histopathological analysis showed that kidneys were abnormal and contained numerous tumor nodules, and these were observed in the interstitial parts of the cortical area of the kidneys. Sections away from the tumor area were also markedly abnormal and demonstrated large dilated congested convoluted tubules, which were lined by somewhat flattened, cuboidal epithelial cells and many of the congested tubules were filled with pink eosinophilic deposits [50]. In the present study on rat kidney, similar results were found with a strong estrogen such as is 17β-estradiol. These studies demonstrated that estrogen can induce abnormal proliferation, principally in the cortical area in rats. However, acute doses of the potent estrogen 17β-estradiol did not produce a tumor but it did induce important damages in the convoluted tubules in rat kidney like in the Syrian hamster model. Other studies suggested that estrogen increased AP-1 transcription factor activity, enhanced the transcription of c-fos and this way the gene activation of type I collagen is inhibited [51,52]. In the present work the inhibition of this type of collagen could be explained by the absence of several glomerular injuries not observed in kidney tissues treated with 17β-estradiol.

The combination of malathion and estrogen treatment caused an increment in glomerular hypertrophy, tubular damage and atypical proliferation in the hilium area after 30 days, and as time passed, the degree of the damage increased. After 124 days, the mixture could produce proliferation in the cortical area, and 240 days post-treatment this group showed serious injuries in kidney tissues with large signs of tubular damage with cell desquamation inside convoluted tubules, high glomeruli hypertrophy, many proliferation zones and in certain cases a malignant proliferation in the hilium zone was found. A direct correlation between oxidative stress and renal dysfunction has been reported [53].

The level of injuries found in kidney tissue suggested an increased oxidative stress caused by malathion treatment in combination with estrogen. Both substances produced several damages in kidneys, not only in glomeruli but also in convoluted tubules. The grade of damage can be serious and can lead to the development of tumors in the urothelium of the hilium. The oxidative stress documented and caused by acute dose of 17β-estradiol, allows us to conclude that the dose used in this study was not sufficient by itself to produce sufficient genomic instability as happened in the hamster model [51,54,55,56,57]. However, estrogen in combination with an organophosphorous pesticide like malathion can produce several injuries in kidney tissue like increases in glomerular hypertrophy, damage in the convoluted tubules, and malignant proliferation in the cortical and hilium areas. It is possible that the oxidative stress caused by malathion exposure enhances the deleterious effects of 17β-estradiol [12,58,59].

Other studies showed that 17β-estradiol in combination with pesticides such as parathion and malathion induced malignant lung and mammary cancer transformations in rats [2,4,60] as well as in *in vitro* studies using the immortalized MCF-10F [61]. Previous *in vitro* experiments showed that estrogen combined with parathion, another organophosphorous pesticide, altered cell proliferation and induced transformation of the MCF-10F cell line [60]. 

Investigations suggest that some chemicals such as pesticides present in the environment, workplace, and home may interact with the estrogen receptors in mammalian cells thereby producing adverse reproductive and developmental effects, and affecting the health of the individual himself and his descendants [33]. Several studies have indicated that malathion could act as an endocrine disruptor, affecting the number and length of each phase of estrus cycle after malathion treatment in rats [62]. 

These studies showed that MGF protein expression was positive in the distal tubules of kidneys of those animals treated with the combination of malathion and estrogen. Since MGF is abundant in breast milk, is synthesized by epithelial cells of differentiated mammary gland and it is present in membranes of the apical portion of these cells, it can serve as a marker for differentiated carcinomas [14]. MFG-E8 is a glycoprotein expressed in many tissues, including mammary glands, and is expressed and often over-expressed on the surface of breast carcinoma cells. [14,15]. The presence of this marker indicates the epithelial origin of the cells. The presence of this marker in convoluted cells and interstitial cells, suggests signs of a metastatic disease since it is absent in the control group. The positive reaction of MGF in convoluted tubules can also be suggested as the result of a colonization of breast cells in kidney tissues. 

Estrogen receptor (ER), including ER-α and ER-β, expression was higher in the presence of the combination of malathion and estrogen when compared to control and the other treatments. The ER pathway is required in the normal growth and physiology of reproductive organs, and it also plays roles in the function of other systems such as the central nervous, skeletal and cardiovascular systems. The antagonistic role of the ER may also promote the development and growth of a variety of cancers and in Syrian hamsters estradiol induced tumor formation and caused kidney cancer [50,63,64,65]. One of the proposed mechanisms by which estrogens induce kidneys tumors is through metabolic conversion of estrogen to catechol metabolites [55]. The oxidation the catechols can give rise to reactive quinones capable of causing DNA damage and redox cycling to generate reactive oxygen species that can cause oxidative damage [65]. This conversion occurs through the cytochrome P450 family, contributing significantly to the metabolic activation of a number of pro-carcinogenic chemicals [66]. It is interesting to note the presence of ERα in some nuclei of distal convoluted tubules and ERβ in collecting tubules. Usually in kidney malignant disease, levels of markers such as ERα, β, PrR and Neu/ErbB2 are negative; however, all animals treated with combination of malathion and estrogen were positive for these receptors [67]. The association of estrogen receptor, progesterone receptor, and human epidermal growth factor receptor 2 (HER2) status of breast cancer patients has been extensively investigated using several approaches. Estrogen receptor negative patients have shown significantly larger tumor mammary tumor volumes, indicating higher angiogenesis with aggressive tumor behavior. The complex molecular mechanism of cell proliferation and the molecular heterogeneity of breast lesions can be also observed in other organs, therefore analysis of kidney tissues in animal treated with combination of malathion and estrogen can be important. The present results suggest an early stage of a possible metastatic process from mammary glands.

Neu/ErbB2 protein expression was higher in malathion-plus estrogen-treated kidneys than control, indicating that these structures have a high degree of malignancy. Previous studies showed that the amplification and over-expression of Neu/ErbB2 is associated with increased progression and metastasis in 25% of human breast carcinomas and is indicative of poor prognosis in breast, ovarian, and renal collecting ductal carcinomas [25,68,69]. Authors have found the co-expression of EGFR and Neu/ErbB2 was also over expressed in transitional cell cancers in ureter or renal pelvis, and that degree of expression was correlated with the histopathological grade of tumor and degree of invasion [70]. 

Interestingly CYP1A1 expression was higher in the presence of combination of malathion and estrogen in comparison to the control group. The activity of this enzyme can be deleterious since it can generate mutagenic metabolites and oxidative stress [71]. The relationship between CYP1A1 activity and susceptibility to chemical carcinogenesis is supported in animal models and also in human populations [72]. The cytochrome CYP1A1 present in extra hepatic tissues is involved in the metabolic activation of several carcinogenic substances [27].

Since PCNA is synthesized during the S phases of the cell cycle [30] it was important to consider whether cells from kidney were affected by the pesticide and estrogen [73]. The combination of malathion and estrogen caused a significant increase in the protein expression of this marker in comparison to control. Since vimentin is a cytoskeletal intermediate filament protein frequently expressed in neoplastic cells with metastatic properties, including breast cancer [31,32], it was interesting to analyze this protein as a marker of metastasis. The combination of malathion and estrogen induced positive protein expression. The high expression of thrombospondin 1 or TSP-1protein observed in kidneys treated with malathion plus estrogen has been associated with wound healing and also with tumor progression of breast cancer metastasis [33,74]. TSP-1 has been shown to promote cell invasion of malignant cells through *in vitro* collagen matrix and metastasis to the lungs [34,35]. It has been suggested that it is probably due to cell invasion by interacting with the extracellular matrix and proteolysis of surrounding cells and detachment of these malignant cells [34,35,74,75]. The dose period of malathion used in these experiments was one sixth the LD_50_ that allowed a 100% survival of animals. The experiments were performed with a 5-day treatment period since we previously showed that malathion induced mammary gland tumors under these conditions [76]. However, our interest this time was to show whether carcinogenesis was induced 240 days after five day treatment in other organs besides mammary glands. The dose of estrogen used in these experiments was lower than the previously reported one since malathion was used in combination in one of the groups and malathion alone induced mammary tumors under these conditions. In previous experiments [77] animals were treated with eserine twice a day for 10 days followed by 17β estradiol for 30 days (200 μg/100 g body weight) once a day, and in another one alone (300 μg/100 g body weight), twice a day for five days. 

## 5. Conclusions

In conclusion, a combination of malathion and estrogen caused phenotypic alterationx in kidney tissues at the protein level. This *in vivo* study is useful to explain why exposure to harmful substances in the environment, such as organophosphorous compounds like malathion may have negative effects on the health of animals and human beings giving us an additional warning against the indiscriminate use of such substances, whose deleterious mixtures are not only a risk factor for renal carcinogenesis but for other tissues like breast, lung and bone marrow.
